# Association between preoperative frailty and myocardial injury after noncardiac surgery in geriatric patients: study protocol for a prospective, multicentre, real-world observational, cohort trial

**DOI:** 10.1186/s12877-024-04847-z

**Published:** 2024-03-19

**Authors:** Yongtao Sun, Na Guo, Min Zhang, Mengjie Liu, Zhongquan Gao, Tao Sun, Xiaojun Gao, Lingling Xu, Haixia Zhang, Chuansong Wei, Peng Liu, Yang Liu, Xiaoning Zhang, Yongle Guo, Lina Chen, Zheng Zhou, Zhenqiang Su, Yanmei Hu, Xin Shi, Linlin Huang, Yuelan Wang

**Affiliations:** 1 Department of Anesthesiology, The First Affiliated Hospital of Shandong First Medical University & Shandong Provincial Qianfoshan Hospital, Shandong Institute of Anesthesia and Respiratory Critical Medicine, Jinan, 250014 China; 2 Department of Anesthesiology, Shandong Provincial Hospital Affiliated to Shandong First Medical University (Shandong Provincial Hospital), Jinan, 250021 China; 3 Department of Anesthesiology, Shandong Public Health Clinical Center, Shandong University, Shandong, 250013 China; 4 Shandong First Medical University & Shandong Academy of Medical Sciences, Jinan, 250117 China; 5 Department of Nursing, The First Affiliated Hospital of Shandong First Medical University & Shandong Provincial Qianfoshan Hospital, Jinan, 250014 China; 6 Department of Clinical Laboratory Medicine, The First Affiliated Hospital of Shandong First Medical University & Shandong Provincial Qianfoshan Hospital, Jinan, 250014 China

**Keywords:** Preoperative, Frailty, Myocardial injury after noncardiac surgery, Geriatric patients, Real world

## Abstract

**Introduction:**

Frailty has become a worldwide health burden that has a large influence on public health and clinical practice. The incidence of frailty is anticipated to increase as the ageing population increases. Myocardial injury after noncardiac surgery (MINS) is associated with short-term and long-term mortality. However, the incidence of MINS in frail geriatric patients is unknown.

**Methods and analysis:**

This prospective, multicentre, real-world observational cohort study will be conducted at 18 designated centres in China from January 2023 to December 2024, with an anticipated sample size of 856 patients aged 65 years and older who are scheduled to undergo noncardiac surgery. The primary outcome will be the incidence of MINS. MINS is defined as a fourth-generation plasma cardiac troponin T (cTnT) concentration ≥ 0.03 ng/mL exhibited at least once within 30 days after surgery, with or without symptoms of myocardial ischaemia. All data will be collected via electronic data acquisition.

**Discussion:**

This study will explore the incidence of MINS in frail patients. The characteristics, predictive factors and 30-day outcomes of MINS in frail patients will be further investigated to lay the foundation for identifying clinical interventions.

**Clinical trial registration:**

https://beta.clinicaltrials.gov/study/NCT05635877, NCT05635877.

**Supplementary Information:**

The online version contains supplementary material available at 10.1186/s12877-024-04847-z.

## Introduction

The ageing population is quickly increasing worldwide, with the number of people aged 65 years and older expected to reach 2 billion by 2050 [1, 2]. The number of geriatric people undergoing surgery over the past 20 years has increased faster than the ageing of the population [3], which has profound implications for the planning and delivery of health and social care. Clinical frailty is the most troubling manifestation of ageing; it is described as a state of increased vulnerability to poor resolution of homeostasis after a stressful event and is the result of a cumulative decline in multiple physiological systems over a lifetime [1, 2]. Many studies have described the prevalence of frailty, and different surgical specialties have different prevalences of frailty. In patients receiving elective orthopaedic surgery, the prevalence of frailty was 23%, whereas in patients undergoing emergency hip fracture surgery, it was 53% [4, 5]. Taking cancer surgery into account, studies have reported that the prevalence of frailty is 25% in patients who undergo elective cystectomy and 39% in patients who undergo emergency general surgery, with the basal lesion usually being a tumour [6, 7]. In vascular surgery, the risk of aortic aneurysm and peripheral artery disease increases with age, and an estimated 52% of patients undergoing elective vascular surgery are frail [8]. Research on rehabilitation and related outcomes has focused on frail geriatric patients during the perioperative period. Frailty is related to adverse perioperative outcomes [9, 10].

In addition to the underlying conditions of sepsis, pulmonary embolism and arrhythmias, MINS is defined as an increase in cardiac troponin (cTn) during noncardiac surgery or within 30 days, with or without ischaemia-related symptoms and signs or myocardial ischaemic changes (with or without necrosis) [11]. Myocardial injury and infarction are considered the leading causes of death after surgery and account for 25% of all postoperative mortalities [12, 13]; ischaemic myocardial injury occurring after surgery but not meeting the criteria for myocardial infarction is more common [14, 15].

Frail populations often have a greater incidence of subclinical changes in cardiac structure and function, which are considered risk factors for the development of cardiovascular diseases (such as hypertension, heart failure and ischaemic heart disease) [16–19]. Many studies have focused on the development of MINS during the perioperative period, and retrospective studies involving adults aged 45 years and older are common. There is currently no pertinent research on the features, risk factors, or 30-day outcomes of MINS in frail geriatric patients. It is urgent to preoperatively assess the comprehensive health status of frail geriatric patients and to reverse or alleviate frailty as soon as possible [20]. Therefore, the main objective of this study will be to investigate the relationship between preoperative frailty and MINS in geriatric patients.

## Methods and analysis

### Study design

This prospective, multicentre, real-world observational cohort trial has been registered with clinicaltrials.gov (version no. YXLL-KY-2022 (089), October 24, 2022) following approval from the First Affiliated Hospital of Shandong First Medical University’s Ethics Committee (NCT05635877). The study will be performed in accordance with the guidelines of good clinical practice and the Declaration of Helsinki [21, 22]. Moreover, this investigation will comply with the standard protocol Item: Intervention Trial Statement Recommendation (SPIRIT) [23].

**Fig. 1:**
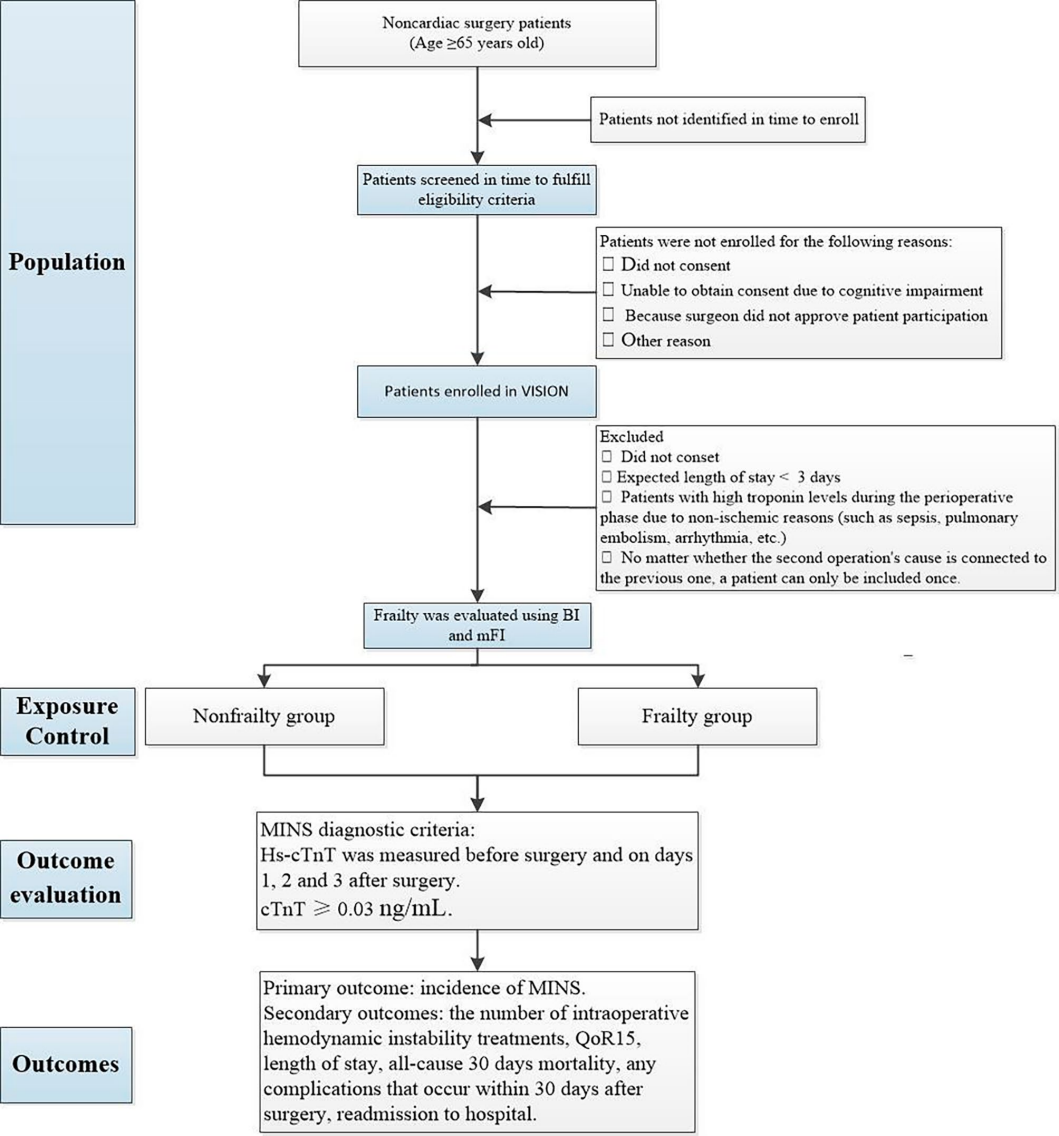
Flow diagram of the study

The trial is being conducted at 18 centres across China (Table 1). The investigation officially began in January 2023, and it is scheduled to be finished in December 2024. The actual start date of the study is January 2023, and the expected completion date is December 2024. There will be a total of 856 participants, of whom 713 will be placed in the non-frailty group and 143 in the frailty group. The study design is shown in Fig. 1.

**Table 1:** Trial centres

Centre	Centre
The First Affiliated Hospital of Shandong First Medical University	Shandong Provincial Hospital Affiliated to Shandong First Medical University
Qilu Hospital of Shandong University	Qilu Hospital of Shandong University (Qingdao)
Affiliated Hospital of Qingdao University	Second Hospital of Shandong University
Chinese PLA 960th Hospital	Qingdao Municipal Hospital
Weihai Municipal Hospital	Liaocheng People’s Hospital
Affiliated Hospital of Jining Medical College	Linyi People’s Hospital
Zibo Central Hospital	Yantai Yuhuangding Hospital
Yantai Affiliated Hospital of Binzhou Medical College	Weifang People’s Hospital
Jining First People’s Hospital	Affiliated Hospital of Weifang Medical College

### Sample size calculation

In this study, the incidence of MINS will be the primary outcome. According to the literature review and pre-experimental results, the incidence of frailty is estimated to be 20%, with a MINS incidence of 15% in the frailty group and 5% in the non-frailty group. A bilateral α = 0.05 will be used with a 90% confidence interval. The sample sizes of the non-frailty and frailty groups have been calculated using the PASS 15.0 program; 570 patients will be included in the non-frailty group, and 114 patients will be included in the frailty group. Considering that the estimated proportion of patients withdrawn or lost to follow-up is 20%, 713 patients will be included in the non-frailty group, and 143 patients will be included in the frailty group; in total, 856 subjects will be included in the study.

### Eligibility criteria

#### Inclusion criteria

- ≥ 65 years of age;
- ASA grades I ∼ IV;
- Elective or emergency surgery;
- Patients undergoing noncardiac surgery;
- Use of general anaesthesia or regional block anaesthesia.

#### Exclusion criteria

- Refusal to provide signed informed consent;
- Expected length of hospital stay < 3 days;
- High perioperative troponin levels due to nonischaemic causes (e.g., sepsis, pulmonary embolism, arrhythmia);
- The same patient can only be included once, regardless of whether the reason for the second operation is related to the first cause.

### Study implementation

Before the study begins, each member of the research team will receive thorough instruction on how to correctly administer the MMSE, aCCI, mFI, BI, EQ-5D-3 L, and QoR15. Participants will only be allowed to participate after passing the test. (Supplemental Appendix 1)

- **Mini-Mental State Exam (MMSE)**. This scale is simple, widely used and the first choice for dementia screening. Time orientation, place orientation, immediate memory, attention and calculation, delayed memory, language, and visual space are the seven components of the scale. There are a total of 30 questions; 1 point is awarded for each correct response, 0 points for a bad response or no response, and the scale’s overall score range is 0 to 30. The test results and educational level are closely associated. Unliterate > 17 points, primary school > 20 points, and junior high and above > 24 points are the typical cut-offs.
- **Age-adjusted Charlson Comorbidity Index (aCCI)**. The ACCI is a more widely used scoring system for complications than the CCI is. Complications are quantified based on the number and severity of patients’ diseases and can be used to predict the risk of disease-related death [24].
- **Assessment of activities of daily living**. The patients will be assessed preoperatively using the Barthel index (BI) evaluation form; 0–40 is classified as severe dysfunction, 41–60 is classified as moderate dysfunction, 61–99 is classified as mild dysfunction, and 100 is classified as self-care.
- **Preoperative frailty assessment** The mFI is an NSQIP-based 11-factor index that has been shown to adequately reflect frailty and predict mortality and morbidity. The mFI is calculated by dividing the number of factors present in a patient by the number of available factors for which there are no missing data. An mFI score of 0 is classified as healthy, 0-0.21 is classified as prefrail, and ≥ 0.21 is classified as prefrail. The index includes 11 items: nonindependent function or activity status, history of diabetes, history of chronic obstructive pulmonary disease (COPD) or pneumonia, history of congestive heart failure, history of myocardial infarction, angina pectoris, percutaneous coronary intervention (PCI), cardiac surgery, hypertension requiring medication, peripheral vascular disease or static pain, sensory disturbance, transient ischaemic attack (TIA) attack or cerebrovascular accident without sequelae, and cerebrovascular accident with sequelae [25]. In this study, patients with moderate or severe dysfunction and a BI ≤ 60 will be considered positive for item 1.
- **European Five-dimensional Three-level Quality of Life Scale (EQ-5D-3 L)**. The EQ-5D scale consists of two parts: the EQ-5D-3 L health description system and the EQ-VAS. The EQ-5D-3 L health description system describes five dimensions: mobility, self-care, usual activities, pain/comfort, and anxiety/depression. Each dimension contains three levels. The EQ-VAS records the interviewees’ self-assessed health status on a vertical visual analogue scale, and the two ends of the scale are marked with “the best health status in your imagination” and “the worst health status in your imagination”. The EQ-VAS provides a quantitative description of respondents’ perceptions of their overall health [26].
- **Diagnostic criteria of MINS**. During noncardiac surgery or within 30 days after surgery, the postoperative troponin level is increased due to myocardial ischaemia (i.e., there is no evidence of nonischaemic causes), and there is no requirement for ischaemic characteristics (such as ischaemic symptoms and ischaemic ECG findings) [27].
- Quality of Recovery-15 (QoR-15**)**. The QoR-15 is a patient-reported outcome measurement validated to measure the QoR after surgery and general anaesthesia. The BDI ranges from 0 to 150, with a higher score indicating better recovery [28]. The QoR-15 is a smaller version of the QoR-40; the psychometric properties are comparable, but the QoR-15 is more practical to use because it is shorter and takes less time to complete [28, 29]. The following QoR scoring criteria will be used: excellent (QoR-15 > 135), good (122 ≤ QoR-15 ≤ 135), moderate (90 ≤ QoR-15 ≤ 121) or poor (QoR-15 **<** 90).

### Exposures, outcomes, and covariates

#### Exposure

- Preoperative frailty in geriatric patients.
- Outcomes of patients followed up 30 days after surgery.

#### Outcomes

**Primary outcome**

- Incidence of MINS. MINS is defined as a peak plasma cTnT concentration of 0.03 ng/mL or greater that is judged to be secondary to myocardial ischaemia (i.e., no evidence of a nonischaemic aetiology causing cTnT elevation, such as sepsis, pulmonary embolism, or myocarditis), which occurs during or within 30 days after surgery. Competent physicians should be informed to further check for ischaemic symptoms, such as myocardial ischaemic symptoms and ECG changes (Supplemental Appendix 1) [13, 30]. Blood samples will be collected before surgery and on the 1st, 2nd and 3rd days after the operation. If a patient has ischaemic symptoms within 30 days after surgery, the researchers will collect blood samples again (Table 2).

**Secondary outcome**

b) Intraoperative haemodynamic instability and treatment times will also be recorded (Supplemental Appendix 2).
c) All-cause mortality within 30 days after the operation (vascular or nonvascular; definitions are provided in Supplemental Appendix 3).
d) Length of stay.
e) Quality of Recovery-15 (QoR-15).
f) Any complications occurred within 30 days after the operation (Supplemental Appendix 4).
g) Readmission to the hospital. (Table 2)

**Table 2:** Study assessment procedures and timetable

	STUDY PERIOD					
	Enrolment	Allocation	Postoperation			Close-out
TIMEPOINT	***-D1***	**0**	***D1***	***D2***	***D3***	***D30***
ENROLMENT						
Eligibility screen	X					
Informed consent	X					
Allocation		X				
ASSESSMENTS						
MMSE	X					
BI	X					
mFI	X					
aCCI	X					
Primary outcome						
MINS		X	X	X	X	X
Secondary outcomes						
QoR-15			X	X	X	
Postoperative complications			X	X	X	X
30-day all-cause mortality			X	X	X	X
30-day readmission			X	X	X	X

MMSE, Mini-Mental State Examination; BI, Barthel Index; mFI, modified frailty index; aCCI, age-adjusted Charlson Comorbidity Index; QoR-15, Quality of Recovery-15.

### Covariant

The main purpose of this study is to determine the incidence of MINS in frail patients. We will include the following baseline covariates to describe the study population and carry out Cox proportional hazard model analysis. The dependent variable is the incidence of MINS within 30 days after noncardiac surgery (using event occurrence time analysis), and the independent variable includes 24 preoperative variables (Supplemental Appendix 5).

### Informed consent

The researchers will obtain consent from the patients before surgery. For patients who cannot provide consent before surgery (such as emergency cases), researchers will obtain consent within 24 h after surgery. Eighteen centres use deferred consent procedures for patients who cannot provide consent (for example, patients taking sedatives and those undergoing mechanical ventilation) and patients who do not have close relatives available [31].

### Sampling

To reflect the seasonal, weekly and daily distributions of the number of surgical cases, patients will be randomly selected according to the methods in the previous study. Each centre is numbered from 0 to 18 in turn, and a computer will be used to generate a series of random numbers. The mantissa corresponds to each centre. At each centre, data will be collected every week or every day. Recruitment should meet two conditions: (1) Patients should be recruited from at least 1–2 centres every day during the study period. (2) For centres with a sample size greater than 20, the interval between two sampling days should be at least 15 days.

### Data management

At each subcentre, investigators will review and approved all the data. The researchers in the participating centres will directly submit the case report forms and supporting documents to the data management system (coordination centre: the First Affiliated Hospital of Shandong First Medical University). The data monitoring of this multicentre study will include a central data consistency check, statistical monitoring and on-site monitoring at all centres.

### Statistical analysis

1. Continuous variables will be expressed as the mean (standard deviation) or median (minimum, maximum; or interquartile interval). The classification variable will be expressed as the number of patients (percentage).
2. A two-sided test will be used for all statistical analyses, and a difference will be statistically significant if the *P* value is less than 0.05.
3. The chi-square test will be used to compare the overall case dropout rate between the two groups.
4. The comparison of baseline numerical variables (such as age) between the groups will be conducted using independent sample t tests or Wilcoxon rank sum tests. Categorical variables (such as sex and the presence of comorbidities) will be compared between the groups by the chi-square test or Fisher’s exact test;
5. The incidence of MINS within 3 days after the operation will be compared using the chi-square test.
6. The incidences of postoperative complications and 30-day mortality will be compared with the chi-square test. The postoperative 30-day survival rate will be calculated by a survival analysis K‒M curve, and the difference between the groups will be evaluated by the log rank test.
7. To determine the postoperative risk of MINS, multivariate logistic regression analysis will be conducted between the baseline and perioperative variables of different groups and the intervention factors, and odds ratios (ORs) and 95% confidence intervals (CIs) will be calculated. According to the main results (the occurrence or absence of MINS), the potential confounding effects on the main results will be adjusted in turn.
8. Survival analysis of the incidence of postoperative MINS in the two groups: K‒M survival curve, Log RANK group comparison, and Cox risk proportion model.

## Discussion

Identifying, treating and preventing frailty has been a major challenge in the field of geriatrics. Due to its multidimensional nature, frailty not only is a strong risk factor for death but also profoundly affects the response, effectiveness and tolerance to drugs and surgical treatment and has a negative impact on quality of life [32–34]. Cardiovascular disease (CVD) is the main cause of death and hospitalization, especially for the geriatric population [18]. Frailty may be preventable, but when it occurs in the stages before death, it may be irreversible. Therefore, strategies to prevent and slow the progression of frailty are crucial [32, 35]. There is an urgent need to study the clinical characteristics, predictive factors and postoperative outcomes of myocardial injury in geriatric frail patients after noncardiac surgery. We will achieve this main purpose through this prospective, multicentre, cohort study.

Due to the use of anaesthetic, sedative and analgesic drugs, perioperative patients may not experience ischaemic symptoms, which leads to failure to identify MINS and postoperative myocardial infarction. Therefore, monitoring hs-cTnT levels during the perioperative period is highly important for identifying the risk of MINS [36]. In 2014, the European Society of Cardiology and the European Society of Anaesthesiology (ESC/ESA) released guidelines that explicitly recommended the clinical use of risk indicators, including myocardial markers, for preoperative risk stratification of patients, and for high-risk patients, cTn detection can be considered before major surgery and within 48–72 h after surgery [6]. The guidelines issued by the Canadian Cardiovascular Society (CCS) in 2017 also recommended that the level of cTn in noncardiac patients be monitored dynamically within 48–72 h after surgery to strengthen the assessment of patients’ perioperative cardiac risk [7]. We hope to clarify the clinical significance of routine detection of cTn levels in geriatric frail patients older than 65 years of age during the perioperative period.

Our research has several limitations. First, this is a nonrandomized controlled trial, so it cannot provide a higher level of evidence than randomized trials. However, as mentioned above, we believe that this is the most appropriate type of research for this issue, and we can draw equally convincing conclusions. Second, the centres we are including are limited to one region, which may lead to heterogeneity. However, whether this conclusion can be extended worldwide is uncertain. However, the large sample size we expect can partially compensate for this problem.

### Electronic supplementary material

Below is the link to the electronic supplementary material.

Supplementary Material 1

## Data Availability

Not applicable.

## Abbreviations

MINS: Myocardial injury after noncardiac surgery
cTnT: cardiac troponin T
cTn: cardiac troponin
MMSE: Mini-Mental State Exam
aCCI: age-adjusted Charlson Comorbidity Index
BI: Barthel index
mFI: modified frailty index
COPD: chronic obstructive pulmonary disease
PCI: percutaneous coronary intervention
TIA: transient ischaemic attack
EQ-5D-3L: European Five-Dimensional Three-Level Quality of Life Scale
QoR15: Quality of recovery-15
ORs: odds ratios
Cis: confidence intervals
CVD: Cardiovascular disease
ESC/ESA: European society of cardiology, European Society of Anaesthesiology
CCS: Cardiovascular Society
