# Supplementary material for: Association between preoperative frailty and myocardial injury after noncardiac surgery in geriatric patients: study protocol for a prospective, multicentre, real-world observational, cohort trial
Source: BMC Geriatr. 2024 Mar 19;24:271. doi: 10.1186/s12877-024-04847-z (PMC10953137; doi:10.1186/s12877-024-04847-z)
Supplement: Supplementary file 1 — Supplementary Material 1 [file 12877_2024_4847_MOESM1_ESM.docx]

**SUPPLEMENTAL MATERIAL**

**Supplemental Appendix 1**. Ischemic symptoms/signs and electrocardiography findings. (1)

1. Ischemic symptoms/signs included any of the following: chest discomfort, arm discomfort, neck discomfort, jaw discomfort, shortness of breath, or pulmonary edema. These ischemic symptoms or signs had to have occurred within 24 hours of an elevated troponin measurement.

2. Ischemic electrocardiography findings included any of the following:

i. development of pathologic Q waves in any two contiguous leads that were ≥30 milliseconds;

ii. development of left bundle branch block (LBBB);

iii. development of ST segment elevation (≥2 mm in leads V1, V2, or V3 OR ≥1 mm in the other leads), ST segment depression (≥1 mm), or symmetric inversion of T waves ≥1 mm in at least two contiguous leads.

ST segment elevation, ST segment depression, and LBBB had to have occurred within 3 days of an elevated troponin measurement, and symmetric T wave inversion had to have occurred within 5 days of an elevated troponin measurement.

**Supplemental Appendix 2.** Definition and treatment of intraoperative hemodynamic instability. (2)

1. **Tachycardia.** Heart rate > 100 BPM if the baseline value > 83 BPM the baseline increased by more than 20 %; diltiazem and / or adjusted anesthetic dose.
2. **Hpertension.** Systolic blood pressure > 160 mmHg, or if the baseline value > 133 mmHg than the baseline increased by more than 20 %; give urapidil or nicardipine and / or adjust the anesthetic dose.
3. **Bradycardia.** Heart rate < 55 BPM, or if the baseline value < 69 BPM than the baseline reduction of more than 20 %; intravenous atropine and / or adjustment of anesthetic dose.
4. **Hypotension**.Systolic blood pressure < 95 mmHg, or if the baseline value < 119 mmHg, it decreased by more than 20 % from the baseline; intravenous infusion of liquid, given vascular compression drugs, and / or adjust the anesthetic dose.

**Supplemental Appendix 3.** Definitions related to cause of death. (3)

1. Vascular death was defined as any death with a vascular cause and included those deaths following a myocardial infarction, cardiac arrest, stroke, cardiac revascularization procedure (i.e., percutaneous coronary intervention [PCI] or coronary artery bypass graft [CABG] surgery), pulmonary embolus, hemorrhage, or deaths due to an unknown cause.

2. Nonvascular death was defined as any death due to a clearly documented non-vascular cause (e.g. trauma, infection, malignancy).

**Supplemental Appendix 4.** Perioperative outcome measures. (2, 4-7)

**Hemorrhage.**

*Wound bleeding*. Local hematomas to be removed.

*Deep bleeding*. Postoperative bleeding needs re-exploration.

**Deep Vein/arterial thrombosis.**

*Deep vein thrombosis.*

*Pulmonary embolism*

When deep vein thrombosis (or arterial embolism) is suspected, it is confirmed by venography or ventilation/flow ratio scan, or by autopsy.

**Cardiovascular complications**

**Heart failure.** Symptoms of left ventricular or congestive heart failure requiring changes in preoperative treatment.

**Arrhythmia.** New postoperative atrial fibrillation, ventricular fibrillation, ventricular tachycardia and other arrhythmias that need to be dealt with.

**Myocardial infarction**. ST-segment elevation or non-elevation, imaging diagnosis.

**Cerebrovascular complications (*Imaging diagnosis*)**

*Hemorrhagic stroke*

*Ischemic stroke*

**Respiratory complications**

**Pulmonary infection**. New sputum or sputum changes, X-ray examination lung texture density changes. Fever, white blood cell count > 12×109/L.

**Respiratory failure**. Postoperative inhalation of air, PaO_2_<60mmHg. PaO_2_:FiO_2_ < 300; Or SPO_2_ < 90% and require oxygen therapy.

**Pleural effusion**. X-ray diagnosis.

**Atelectasis.** X-ray diagnosis.

**Pneumothorax.** X-ray diagnosis.

**Bronchospasm.** New end expiratory wheezing that requires only bronchodilator medication.

**Aspiration pneumonia.** Acute lung injury caused by inhalation of gastric reflux.

With at least one positive.

1. No other cause of fever > 38 °C
2. White blood cell 12 × 10^9^ / L
3. Change of consciousness in patients over 70 without other causes.

Compound at least 2 positive in the following.

1. New sputum / sputum changes
2. Excretion increase / sputum suction
3. New cough / aggravated cough / dyspnea / shortness of breath
4. Loudness / Bronchial breathing sound
5. Deterioration of gas exchange state.

**Acute Respiratory Distress Syndrome ARDS.** Mechanical ventilation, X-ray showed patchy infiltration of bilateral lungs; PaO_2_:FiO_2_ < 300, and no evidence of left atrial overload within 7 days after surgery.

**Tracheobronchitis.** Chest radiographs are normal with thick sputum.

**Pulmonary edema.** Pulmonary congestion or stasis, acute pulmonary edema, congestive heart failure, excessive infusion.

**Lung disease has worsened.**

**Gastrointestinal Complications**

**Indigestion.** Bloating, constipation.

**Post-operative stres**s ulcer. Gastrointestinal bleeding.

**Acute kidney injury**

Serum creatinine increased ≥ 0.3mg / dl ( ≥ 26.5μmol / l ) within 48h. and

Predicted or actual serum creatinine increase ≥ 1.5 times baseline in the past 7 days. and

Urine volume < 0.5ml /kg/h, more than 6h.

Kidney Disease Improving Global Outcomes Guidelines (KIDGO)

| Stage | Serum creatinine | Urine output |
| --- | --- | --- |
| 1 | 1.5–1.9 times baseline value within 7 days | ≤ 0.5 mL kg^-1^ h^-1^ for 6–12 h |
|  | or |  |
|  | ≥ 27mmol L^-1^ (0.3 mg dL^-1^) increase within 48 h |  |
| 2 | 2.0–2.9 times baseline value within 7 days | ≤ 0.5 mL kg^-1^ h^-1^ for ≥12 h |
| 3 | ≥ 3.0 times baseline within 7 days | Urine volume < 0.3 ml/kg/h for ≥ 24 h |
|  | or | or |
|  | Increase in serum creatinine to ≥ 354 mmol L^-1^ (≥ 4.0 mg dL^-1^ with an acute rise of > 44 mmol L^-1^ (0.5 mg/dL^-1^) | No urine ≥ 12h |
|  | or |  |
|  | Initiation of renal replacement therapy |  |
|  | or |  |
|  | In patients < 18 years, decrease in eGFR to < 35 mL min^-1^ per 1.73 m^2^ |  |

**Sepsis**

Life-threatening condition in which the host's immune response to infection attacks its own organs.

**Other infection，not sepsis**

Body temperature > 38 °C or < 36 °C, white blood cell < 4 × 10^9^ L or > 12 × 10^9^ / L, increased C-reactive protein, procalcitonin or IL-6.

**Blood culture positive for bacteria.**

**Wounds.** Wound cellulitis or purulent secretions.

**Urine.** Patients with clear urine had positive white blood cells and bacteria content > 105 / ml.

**Deep infection.** Clinical or imaging diagnosis of intraperitoneal infection.

**Fever of unknown origin.** After recovery from postoperative primary fever (if applicable), the body temperature exceeded 37 ° C for more than 24 hours and no definite cause was found.

**Second operation needed**

Patients requiring secondary surgery during hospitalization.

**Supplemental Appendix 5.** Preoperative characteristics and definitions of patients. (1, 3)

1. **Age** – Patient age in years was recorded and subsequently evaluated as: i. 65-79 years of age; and ii. ≥80 years of age.
2. **Sex –** Male or female.
3. **Body mass index (BMI)** – i. BMI＜18, ii. BMI 18.0-27.9, ⅲ. BMI ≥ 28kg/m^2^.
4. **History of coronary artery disease** – A current or prior history of any one of the following: i. angina; ii. myocardial infarction or acute coronary syndrome; iii. a segmental cardiac wall motion abnormality on echocardiography or a segmental fixed defect on radionuclide imaging; iv. a positive radionuclide exercise, echocardiographic exercise, or pharmacological cardiovascular stress test demonstrating cardiac ischemia; v. coronary angiographic or CT coronary angiographic evidence of atherosclerotic stenosis ≥50% of the diameter of any coronary artery; vi. electrocardiogram with pathological Q waves in two contiguous leads.
5. **History of cardiac arrest** – A patient with a prior history of a cardiac arrest.
6. **History of congestive heart failure** – A physician diagnosis of a current or prior episode of congestive heart failure or prior radiographic evidence of vascular redistribution, interstitial pulmonary edema, or frank alveolar pulmonary edema.
7. **History of peripheral vascular disease** – A physician diagnosis of a current or prior history of intermittent claudication, vascular surgery for atherosclerotic disease, an ankle/arm systolic blood pressure ratio ≤ 0.90 in either leg at rest, or angiographic or doppler study demonstrating ≥ 70% stenosis in a noncardiac artery.
8. **History of deep vein thrombosis (DVT) or pulmonary embolus (PE)** - Patients with a current or previous history of DVT or PE.
9. **History of stroke** – A physician diagnosis of a current or prior stroke, or CT or magnetic resonance evidence of a stroke.
10. **History of transient Ischemic Attack (TIA)** - A physician's diagnosis of a current or past TIA.
11. **Diabetes** – Patient stated that they have a diagnosis of diabetes or a physician has previously recorded that the patient has diabetes. This included gestational diabetes at the time of noncardiac surgery, but not past gestational diabetes that had resolved.
12. **Hypertension** – A physician diagnosis of hypertension.
13. **Current atrial fibrillation** – A patient with a current history of atrial fibrillation.
14. **Chronic obstructive pulmonary disease (COPD)** – A physician current or prior diagnosis of chronic bronchitis, emphysema, or COPD, or a patient provided a history of daily production of sputum for at least 3 months in 2 consecutive years.
15. **Obstructive sleep apnea (OSA)** - Obstructive sleep apnea is diagnosed by a physician or sleep study.
16. **Active cancer** – A patient was designated as having active cancer if they fulfilled any of the following criteria: i. undergoing surgery for cancer; ii. known metastatic disease; or iii. patient had received active treatment for their cancer (e.g., chemotherapy, radiation, or surgery) within the 6 months prior to their surgery, but this did not apply to patients with non-melanoma skin cancers or surgery for a biopsy.
17. **Urgent/Emergency surgery** – Emergency surgery was surgery that occurred <24 hours after a patient developed an acute surgical condition, and urgent surgery was surgery that occurred 24-72 hours after a patient developed an acute surgical condition.
18. **Major orthopedic surgery** – A patient undergoing one or more of the following orthopedic surgeries: major hip or pelvis surgery, internal fixation of femur, knee arthroplasty, above knee amputations, or lower leg amputation (amputation below knee but above foot).
19. **Major general surgery** – A patient undergoing one or more of the following general surgeries: complex visceral resection, partial or total colectomy or stomach surgery, other intra-abdominal surgery, or major head and neck resection for non-thyroid tumor.
20. **Major urology or gynecology surgery** – A patient undergoing one or more of the following major urology or gynecology surgeries: nephrectomy, ureterectomy, bladder resection, retroperitoneal tumor resection, exenteration, cytoreduction surgery, hysterectomy, radical prostatectomy, or transurethral prostatectomy.
21. **Major neurosurgery** – A patient undergoing one or more of the following neurosurgeries: craniotomy or major spine surgery (i.e., surgery involving multiple levels of the spine).
22. **Major vascular surgery** – A patient undergoing one or more of the following vascular surgeries: thoracic aorta reconstructive vascular surgery, aorto-iliac reconstructive vascular surgery, peripheral vascular reconstruction without aortic cross-clamping, extracranial cerebrovascular surgery, or endovascular abdominal aortic aneurysm repair.
23. **Major thoracic surgery** – A patient undergoing one or more of the following thoracic surgeries: pneumonectomy, lobectomy, wedge resection of lung, resection of mediastinal tumor, or major chest wall resection.
24. **Low-risk surgeries** – A patient undergoing one or more of the following surgeries: parathyroid, thyroid, breast, hernia, local anorectal procedure, oopherectomy, salpingectomy, endometrial ablation, peripheral nerve surgery, ophthalmology, ears/nose/throat surgery, vertebral disc surgery, hand surgery, cosmetic surgery, arterio-venous access surgery for dialysis, or any other surgery not mentioned above.

**References**

1. F. Botto, P. Alonso-Coello, M. T. Chan, J. C. Villar, D. Xavier, S. Srinathan, G. Guyatt, P. Cruz, M. Graham, C. Y. Wang, O. Berwanger, R. M. Pearse, B. M. Biccard, V. Abraham, G. Malaga, G. S. Hillis, R. N. Rodseth, D. Cook, C. A. Polanczyk, W. Szczeklik, D. I. Sessler, T. Sheth, G. L. Ackland, M. Leuwer, A. X. Garg, Y. Lemanach, S. Pettit, D. Heels-Ansdell, G. Luratibuse, M. Walsh, R. Sapsford, H. J. Schünemann, A. Kurz, S. Thomas, M. Mrkobrada, L. Thabane, H. Gerstein, P. Paniagua, P. Nagele, P. Raina, S. Yusuf, P. J. Devereaux, P. J. Devereaux, D. I. Sessler, M. Walsh, G. Guyatt, M. J. McQueen, M. Bhandari, D. Cook, J. Bosch, N. Buckley, S. Yusuf, C. K. Chow, G. S. Hillis, R. Halliwell, S. Li, V. W. Lee, J. Mooney, C. A. Polanczyk, M. V. Furtado, O. Berwanger, E. Suzumura, E. Santucci, K. Leite, J. A. Santo, C. A. Jardim, A. B. Cavalcanti, H. P. Guimaraes, M. J. Jacka, M. Graham, F. McAlister, S. McMurtry, D. Townsend, N. Pannu, S. Bagshaw, A. Bessissow, M. Bhandari, E. Duceppe, J. Eikelboom, J. Ganame, J. Hankinson, S. Hill, S. Jolly, A. Lamy, E. Ling, P. Magloire, G. Pare, D. Reddy, D. Szalay, J. Tittley, J. Weitz, R. Whitlock, S. Darvish-Kazim, J. Debeer, P. Kavsak, C. Kearon, R. Mizera, M. O'Donnell, M. McQueen, J. Pinthus, S. Ribas, M. Simunovic, V. Tandon, T. Vanhelder, M. Winemaker, H. Gerstein, S. McDonald, P. O'Bryne, A. Patel, J. Paul, Z. Punthakee, K. Raymer, O. Salehian, F. Spencer, S. Walter, A. Worster, A. Adili, C. Clase, D. Cook, M. Crowther, J. Douketis, A. Gangji, P. Jackson, W. Lim, P. Lovrics, S. Mazzadi, W. Orovan, J. Rudkowski, M. Soth, M. Tiboni, R. Acedillo, A. Garg, A. Hildebrand, N. Lam, D. Macneil, M. Mrkobrada, P. S. Roshanov, S. K. Srinathan, C. Ramsey, P. S. John, L. Thorlacius, F. S. Siddiqui, H. P. Grocott, A. McKay, T. W. Lee, R. Amadeo, D. Funk, H. McDonald, J. Zacharias, J. C. Villar, O. L. Cortés, M. S. Chaparro, S. Vásquez, A. Castañeda, S. Ferreira, P. Coriat, D. Monneret, J. P. Goarin, C. I. Esteve, C. Royer, G. Daas, M. T. Chan, G. Y. Choi, T. Gin, L. C. Lit, D. Xavier, A. Sigamani, A. Faruqui, R. Dhanpal, S. Almeida, J. Cherian, S. Furruqh, V. Abraham, L. Afzal, P. George, S. Mala, H. Schünemann, P. Muti, E. Vizza, C. Y. Wang, G. S. Ong, M. Mansor, A. S. Tan, Shariffuddin, II, V. Vasanthan, N. H. Hashim, A. W. Undok, U. Ki, H. Y. Lai, W. A. Ahmad, A. H. Razack, G. Malaga, V. Valderrama-Victoria, J. D. Loza-Herrera, M. De Los Angeles Lazo, A. Rotta-Rotta, W. Szczeklik, B. Sokolowska, J. Musial, J. Gorka, P. Iwaszczuk, M. Kozka, M. Chwala, M. Raczek, T. Mrowiecki, B. Kaczmarek, B. Biccard, H. Cassimjee, D. Gopalan, T. Kisten, A. Mugabi, P. Naidoo, R. Naidoo, R. Rodseth, D. Skinner, A. Torborg, P. Paniagua, G. Urrutia, M. L. Maestre, M. Santaló, R. Gonzalez, A. Font, C. Martínez, X. Pelaez, M. De Antonio, J. M. Villamor, J. A. García, M. J. Ferré, E. Popova, P. Alonso-Coello, I. Garutti, P. Cruz, C. Fernández, M. Palencia, S. Díaz, T. Del Castillo, A. Varela, A. de Miguel, M. Muñoz, P. Piñeiro, G. Cusati, M. Del Barrio, M. J. Membrillo, D. Orozco, F. Reyes, R. J. Sapsford, J. Barth, J. Scott, A. Hall, S. Howell, M. Lobley, J. Woods, S. Howard, J. Fletcher, N. Dewhirst, C. Williams, A. Rushton, I. Welters, M. Leuwer, R. Pearse, G. Ackland, A. Khan, E. Niebrzegowska, S. Benton, A. Wragg, A. Archbold, A. Smith, E. McAlees, C. Ramballi, N. Macdonald, M. Januszewska, R. Stephens, A. Reyes, L. G. Paredes, P. Sultan, D. Cain, J. Whittle, A. G. Del Arroyo, D. I. Sessler, A. Kurz, Z. Sun, P. S. Finnegan, C. Egan, H. Honar, A. Shahinyan, K. Panjasawatwong, A. Y. Fu, S. Wang, E. Reineks, P. Nagele, J. Blood, M. Kalin, D. Gibson and T. Wildes: Myocardial injury after noncardiac surgery: a large, international, prospective cohort study establishing diagnostic criteria, characteristics, predictors, and 30-day outcomes. *Anesthesiology*, 120(3), 564-78 (2014) doi:10.1097/aln.0000000000000113

2. A. Miskovic and A. B. Lumb: Postoperative pulmonary complications. *Br J Anaesth*, 118(3), 317-334 (2017) doi:10.1093/bja/aex002

3. P. J. Devereaux, M. T. Chan, P. Alonso-Coello, M. Walsh, O. Berwanger, J. C. Villar, C. Y. Wang, R. I. Garutti, M. J. Jacka, A. Sigamani, S. Srinathan, B. M. Biccard, C. K. Chow, V. Abraham, M. Tiboni, S. Pettit, W. Szczeklik, G. Lurati Buse, F. Botto, G. Guyatt, D. Heels-Ansdell, D. I. Sessler, K. Thorlund, A. X. Garg, M. Mrkobrada, S. Thomas, R. N. Rodseth, R. M. Pearse, L. Thabane, M. J. McQueen, T. VanHelder, M. Bhandari, J. Bosch, A. Kurz, C. Polanczyk, G. Malaga, P. Nagele, Y. Le Manach, M. Leuwer and S. Yusuf: Association between postoperative troponin levels and 30-day mortality among patients undergoing noncardiac surgery. *Jama*, 307(21), 2295-304 (2012) doi:10.1001/jama.2012.5502

4. G. P. Copeland, D. Jones and M. Walters: POSSUM: a scoring system for surgical audit. *Br J Surg*, 78(3), 355-60 (1991) doi:10.1002/bjs.1800780327

5. P. Carpintero, J. R. Caeiro, R. Carpintero, A. Morales, S. Silva and M. Mesa: Complications of hip fractures: A review. *World J Orthop*, 5(4), 402-11 (2014) doi:10.5312/wjo.v5.i4.402

6. Summary of Recommendation Statements. *Kidney Int Suppl (2011)*, 2(1), 8-12 (2012) doi:10.1038/kisup.2012.7

7. M. Singer, C. S. Deutschman, C. W. Seymour, M. Shankar-Hari, D. Annane, M. Bauer, R. Bellomo, G. R. Bernard, J. D. Chiche, C. M. Coopersmith, R. S. Hotchkiss, M. M. Levy, J. C. Marshall, G. S. Martin, S. M. Opal, G. D. Rubenfeld, T. van der Poll, J. L. Vincent and D. C. Angus: The Third International Consensus Definitions for Sepsis and Septic Shock (Sepsis-3). *Jama*, 315(8), 801-10 (2016) doi:10.1001/jama.2016.0287
